# Unraveling the differential mechanisms of revascularization promoted by MSCs & ECFCs from adipose tissue or umbilical cord in a murine model of critical limb-threatening ischemia

**DOI:** 10.1186/s12929-024-01059-w

**Published:** 2024-07-15

**Authors:** Marta Rojas-Torres, Lucía Beltrán-Camacho, Ana Martínez-Val, Ismael Sánchez-Gomar, Sara Eslava-Alcón, Antonio Rosal-Vela, Margarita Jiménez-Palomares, Esther Doiz-Artázcoz, Mario Martínez-Torija, Rafael Moreno-Luna, Jesper V. Olsen, Ma. Carmen Duran-Ruiz

**Affiliations:** 1 Biomedicine, Biotechnology and Public Health Department, University of Cadiz, Cadiz, 11002 Spain; 2 Biomedical Research and Innovation Institute of Cadiz (INiBICA), Cadiz, 11002 Spain; 3 Cell Biology, Physiology and Immunology Department, University of Cordoba, Cordoba, 14004 Spain; 4 Maimonides Biomedical Research Institute of Cordoba (IMIBIC), Cordoba, 14004 Spain; 5 National Center of Cardiovascular Research Carlos III (CNIC), Madrid, 28029 Spain; 6 Angiology & Vascular Surgery Unit, Hospital Universitario Puerta del Mar, Cadiz, Spain; 7 Pathophysiology and Regenerative Medicine Group, Hospital Nacional de Parapléjicos (SESCAM), Toledo, 45071 Spain; 8 Nursing department, Hospital Universitario de Toledo (SESCAM), Toledo, 45071 Spain; 9 Cooperative Research Network Orientated to Health Results, Vascular Brain Diseases, RICORS-ICTUS, SESCAM, Toledo, Spain; 10 Novo Nordisk Foundation Center for Protein Research, Copenhagen, Denmark; 11 Biomedicine, Biotechnology and Public Health Department, Science Faculty, Cádiz University. Torre Sur. Avda. República Saharaui S/N, Polígono Río San Pedro, Puerto Real, Cádiz, 11519 Spain

**Keywords:** Cell therapy, MSCs, ECFCs, Adipose tissue, Critical limb-threatening ischemia, Proteomics

## Abstract

**Background:**

Critical limb-threatening ischemia (CLTI) constitutes the most severe manifestation of peripheral artery disease, usually induced by atherosclerosis. CLTI patients suffer from high risk of amputation of the lower extremities and elevated mortality rates, while they have low options for surgical revascularization due to associated comorbidities. Alternatively, cell-based therapeutic strategies represent an effective and safe approach to promote revascularization. However, the variability seen in several factors such as cell combinations or doses applied, have limited their success in clinical trials, being necessary to reach a consensus regarding the optimal “cellular-cocktail” prior further application into the clinic. To achieve so, it is essential to understand the mechanisms by which these cells exert their regenerative properties.

Herein, we have evaluated, for the first time, the regenerative and vasculogenic potential of a combination of endothelial colony forming cells (ECFCs) and mesenchymal stem cells (MSCs) isolated from adipose-tissue (AT), compared with ECFCs from umbilical cord blood (CB-ECFCs) and AT-MSCs, in a murine model of CLTI.

**Methods:**

Balb-c nude mice (n:32) were distributed in four different groups (n:8/group): control shams, and ischemic mice (after femoral ligation) that received 50 µl of physiological serum alone or a cellular combination of AT-MSCs with either CB-ECFCs or AT-ECFCs. Follow-up of blood flow reperfusion and ischemic symptoms was carried out for 21 days, when mice were sacrificed to evaluate vascular density formation. Moreover, the long-term molecular changes in response to CLTI and both cell combinations were analyzed in a proteomic quantitative approach.

**Results:**

AT-MSCs with either AT- or CB-ECFCs, promoted a significant recovery of blood flow in CLTI mice 21 days post-ischemia. Besides, they modulated the inflammatory and necrotic related processes, although the CB group presented the slowest ischemic progression along the assay. Moreover, many proteins involved in the repairing mechanisms promoted by cell treatments were identified.

**Conclusions:**

The combination of AT-MSCs with AT-ECFCs or with CB-ECFCs promoted similar revascularization in CLTI mice, by restoring blood flow levels, together with the modulation of the inflammatory and necrotic processes, and reduction of muscle damage. The protein changes identified are representative of the molecular mechanisms involved in ECFCs and MSCs-induced revascularization (immune response, vascular repair, muscle regeneration, etc.).

**Supplementary Information:**

The online version contains supplementary material available at 10.1186/s12929-024-01059-w.

## Introduction

Peripheral arterial disease (PAD) is a cardiovascular atherosclerotic disease which results from the narrowing and obstruction of major systemic arteries different from those of the cerebral and coronary circulations, although is mainly associated to the lower extremities. Despite PAD initially courses as asymptomatic, many patients progress during adulthood to chronic limb-threatening ischemia (CLTI), the most severe manifestation of PAD, which entails a high risk of amputation and elevated mortality rates [1]. CLTI patients suffer from chronic rest pain, ischemic ulcers and eventual amputation of toes and extremities [2]. Moreover, CLTI prognosis is frequently worsened with the presence of risk factors that aggravate vascular damage, such as diabetes, hyperlipidemia, or smoking habits [3, 4]. Nowadays, the standard therapy for these patients is surgical revascularization, however, only 30% are suitable for this procedure [5], due to related comorbidities as well as surgical-related difficulties [6, 7]. In this sense, angiogenic therapy and in particular, cell-based therapy, have been explored as a promising treatment for CLTI patients, mainly for those with no option for surgical or endovascular revascularization [8, 9].

In the last decade, adult bone marrow (BM) was considered the major source of stem or progenitor cells, as they comprise, apart from mesenchymal stem cells (MSCs), a subset of endothelial progenitor cells (EPCs) and angioblasts, which are beneficial for vasculogenesis and neovascularization [10]. Since the first autologous implantation in 2002 in CLTI patients [9], numerous pre-clinical and clinical studies have reported the beneficial effects of different combinations of BM-derived cells [5]. However, while BM aspiration constitutes an invasive procedure associated with risks of infection, bleeding, and pain, alternative sources currently used such as umbilical cord blood (CB) or adipose tissue (AT), are less susceptible of viral contamination and present an easier and non-invasive access [11, 12]. Derived from these sources, different cell types have been tested, alone or in combination, in both clinical and pre-clinical studies, including, BM-derived mononuclear cells (BM-MNCs), EPCs, MSCs, and other tissue-derived progenitors [4, 9, 13–15]. Among them, endothelial colony-forming cells (ECFCs) are precursors committed to the endothelial lineage with robust vasculogenic properties [16, 17]. MSCs, on the other hand, are multipotent and fibroblast-like adherent cells that can differentiate into various cell types such as bone cartilage, fat, and muscle. Their therapeutic effect relies mainly on the secretion of paracrine factors with angiogenic, anti-apoptotic, anti-inflammatory, and immunomodulatory effects [18]. Moreover, the combined administration of MSCs with ECFCs has been postulated as an effective treatment for vascular regenerative medicine. When administered together, MSCs improve ECFCs engraftment and function, while they also modulate the immune response towards these cells [19]. Thus, most studies have used BM-derived MSCs combined with ECFCs isolated from either mononuclear cells (MNC) from human adult blood, or from human CB. While CB-ECFCs have been reported to restore blood perfusion in hind limb ischemia (HLI) [20, 21], they originate from non-adult tissues, complicating their use in general clinical practice. On the other hand, AT-derived ECFCs share similar properties to those obtained from adult peripheral blood, including a robust ability to form functional blood vessels in vivo [22]. AT-ECFCs regenerative properties have been tested alone or combined with MSCs in vitro and in xenograft implants [23], but no one, to our knowledge, has analyzed their effect in a CLTI model.

In the current study we have evaluated, for the first time, the regenerative and vasculogenic potential of AT-derived cells, AT-ECFCs and AT-MSCs, in a murine model of CLTI, comparing this effect with the one produced by administration of CB-ECFCs and AT-MSCs. Furthermore, the long-term molecular changes seen in CLTI and in response to both combinations have been evaluated in a proteomic quantitative approach, in order to understand the mechanisms potentially involved in ECFCs and MSCs induced revascularization within the ischemic tissues.

## Methods

### Cell isolation and culture of ECFCs and MSCs

Adipose tissue (AT) cells (both AT-ECFCs and AT-MSCs) were isolated from normal subcutaneous white adipose tissue (s-WAT) from healthy donors (aged between 55 and 65 years old), provided after routine medical procedures at the Hospital Nacional de Parapléjicos, adhering to ethically approved protocols. Briefly, 5 gr of s-WAT samples underwent enzymatic digestion with collagenase and dispase at 37ºC for 1.5 h, followed by centrifugation (2500 g, 10 min) to isolate the pellet post-removal of mature adipocytes. Cell pellets were resuspended in D10 medium, comprising DMEM (high glucose) supplemented with 10% FBS, 1% glutamine, and penicillin–streptomycin (GPS; Invitrogen, Carlsbad, CA), and filtered through a 100-µm cell strainer (Fisherbrand). Subsequently, AT-ECFCs and AT-MSCs were purified using a previously established protocol [22], with slight adaptations. AT-ECFCs were isolated via magnetic-activated cell sorting (MACS) utilizing CD31-coated magnetic beads (Dynalbeads; Invitrogen, Grand Island, NY) and cultured on gelatin-coated plates using ECFC medium: EGM-2 (excluding hydrocortisone; Lonza, Walkersville, MD) supplemented with 20% FBS, and GPS. AT-MSCs were obtained from the CD31-negative fraction of AT-ECFCs and cultured on coated plates using MSC medium: MSCGM-2 (Promocells), supplemented with 10% FBS and 1% GPS.

Human embryonic derived ECFCs (CB-ECFCs) were isolated from human umbilical cord blood as described [24, 25]. Thus, mononuclear cells from CB were seeded on fibronectin-coated plates (Corning- Biocoat) and cultivated with the same medium used for AT-ECFCs. Endothelial colonies were identified as cohesive monolayers of > 50 cells with cobblestone morphology.

### Cell characterization

The expansion potential of ECFCs was systematically evaluated through standardized passage protocols. To achieve so, each cell type was seeded on gelatin-coated plates with medium replenishment every 2–3 days. AT-ECFCs and CB-ECFCs were seeded at 15000 cells /cm^2^ using ECFC medium over a 32-day expansion period, while AT-MSCs were seeded at 10000 cells/cm^2^ using MSC medium over a 28-day expansion period. Harvesting was conducted via trypsinization, with subsequent passages involving reseeding of harvested cells under identical conditions. Cumulative cell counts were determined at the end of each passage using a hemocytometer. At each expansion passage, 10^6^ cells were cryopreserved and stored in liquid nitrogen for future use. All cell populations were used between passages 5 and 7.

Cell identity of ECFCs was confirmed by testing cloning-forming ability, together with the validation of several surface markers by flow cytometry, as previously described [26–28], using the Cytoflex cytometer (Beckman Coulter) and CytoExpert software. The full list of antibodies can be found in Supplementary Table S1. Similarly, MSCs identity was validated by performing ex vivo multilineage differentiation protocols, testing the ability of MSCs to differentiate to adipocytes, assessed by Oil Red staining, osteocytes and chondrocytes, as described [23].

### Animals

Male Balb-C Nude (CAnN.Cg-*Foxn1*^*nu*^/Crl) mice (n:32), age 9 weeks, were obtained from Charles River Laboratories. Animals were allocated in special rooms, where they were fed sterile standard chow diet ad libitum*,* and had free access to sterile water. Technical staff were constantly supervising filters and air recirculation and monitoring any signs of ill-health. Finally, mice were euthanized in a CO_2_ chamber at the end of the assay.

### CLTI murine model and evaluation of the regenerative effect

Balb-c nude mice (n:24) were anesthetized before surgery with ketamine (100 mg/kg) and xylazine (10 mg/kg) by subcutaneous administration, and a double ligation of the left femoral artery (FAL) was then performed, occluding both distal and proximal ends with double knots of suture (non-absorbable 6/0), as described [5, 29–31]. Mice received analgesic injected intraperitoneally (Ketoprofen, 2 mg/kg) for three consecutive days.

Animals were equally distributed between groups, based on blood flow ratios and ischemic status registered 24 h after surgery, before cell and saline serum administration. Mice received an intramuscular injection of 50 µl of physiological serum without cells, as ischemic controls (IC, n:8) or 50 µl of physiological serum containing either AT-ECFCs (1.2·10^6^) and AT-MSCs (8·10^5^) (AT; n:8) or CB-ECFCs (1.2·10^6^) and AT-MSCs (8·10^5^) (CB, n:8). Cell administration was performed in 3–4 different sites of the left limb muscle: low back, low frontal, and middle muscles. In addition, sham surgical controls (SH, n:8) were employed for vascular density changes, immune cell detection and proteomic assays. A full description of FAL procedure and the injection sites can be found in Supplementary Figure S1.

### Follow-up of physiological changes after CLTI surgery and cell administration

**Fig. 1:**
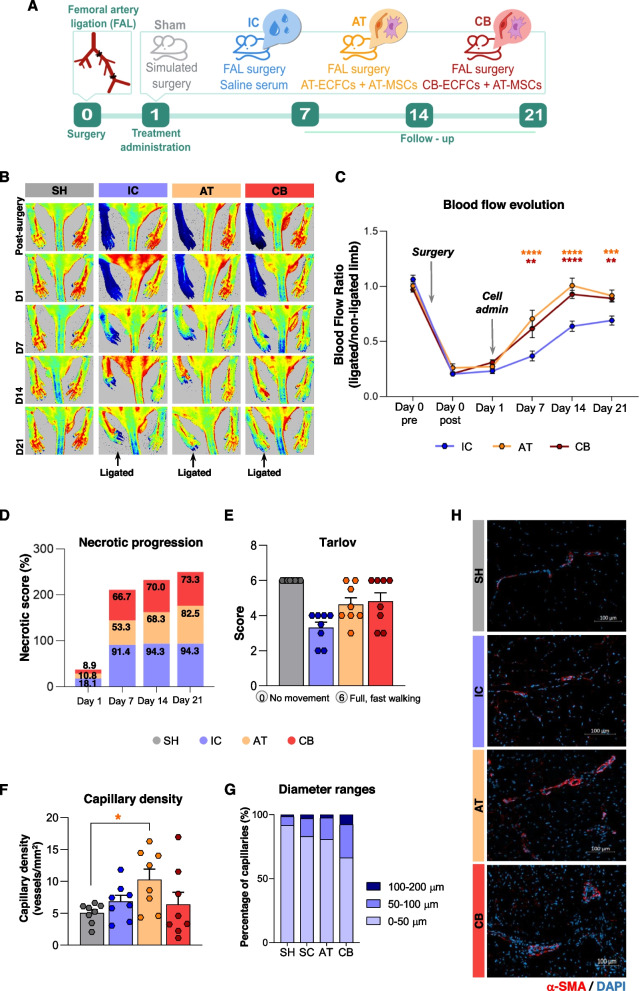
Experimental workflow and follow-up of ischemic symptoms in CLTI mice in response to cell administration. **A** Schematic representation of the experimental assay. Mice underwent femoral artery ligation (FAL, biological replicates or BR, n:24) or simulated surgery (Sham group, n:8 BR), and 24 h after, they were administered saline serum (ischemic untreated control, IC group, n:8), AT-ECFCs and MSCs (AT group, n:8) or CB-ECFCs and AT-MSCs (CB group, n:8). Follow-up of ischemic symptoms was done on day 1, 7, 14, and 21 after surgery (n:32). **B** Representative laser Doppler images from all groups of blood flow (BF) measurements using Pericam PSI HR system: post-surgery, on day 1, day 7, day 14, and day 21 (BR n:32). **C** Graphical representation of BF changes per group within time. Perfusion (PU) averaged ratios of left (ligated) *vs.* right (non-ligated) limbs are shown (BR: IC, n:8; AT, n:8; CB, n:8). **D** Necrosis progression, represented as the percentage of necrotic tissue affected per group, registered on days 1, 7, 14, and 21 BR: IC, n:8; AT, n:8; CB, n:8). **E** Motility changes (Tarlov score) registered at day 21 (BR: SH, n:8; IC, n:8; AT, n:8; CB, n:8). **F** Capillary density, the number of blood vessels (vessels/mm^2^) was measured by staining left limb muscles from all groups (BR: SH, n:8; IC, n:8; AT, n:8; CB, n:8; technical replicates, n:3). **G** Vessel classification based on abundance percentage of different ranges of internal lumen diameter (μm) (BR: SH, n:8; IC, n:8; AT, n:8; CB, n:8; technical replicates, n:3). Data were presented as mean ± SEM. Statistically significant differences after performing Two-way ANOVA and Tukey’s as post-hoc (**C**) or Kruskal Wallis and post-hoc Dunn’s test (**F**) are shown as * *p*-value < 0.05, ** *p*-value < 0.01, *** *p*-value < 0.001. I). **H** Representative images obtained by immunohistochemistry, used to measure vascular density and diameter size, using anti-mouse smooth muscle α-actin (α-SMA) antibody (red) and DAPI (blue)

Blood flow (BF) was measured for both paws at baseline (prior surgery, day 0 pre-), right after surgery (day 0 post-) and at days 1, 7, 14, and 21 using a PeriCam PSI HR system (Perimed; Järfälla, Sweden). An overview of the workflow is shown in Fig. 1.

During the procedure, animals were kept anesthetized with ketamine (100 mg/kg) and xylazine (10 mg/kg), placed under the camera in a pad with controlled temperature (36.5–37.5ºC). Perfusion was expressed as the ratio of left (ischemic) *vs* right (non-ischemic) limb, and blood flow values registered within anatomically defined regions of interest (ROIs). In parallel, motility impairment, inflammation, ulceration, and necrosis were also registered for all mice during the entire assay according to Tarlov [32] and ischemia scores [33], as described [5, 29, 30] (Supplementary Table S2). In order to get closer to the necrotic progression seen in our CLTI animal model, we defined a necrotic score based on the number of necrotic nails, fingers or paw. Briefly, a score of 0.5 was assigned to each necrotic nail, while a punctuation of 1 was assigned additionally if the finger was also necrotic compromised. Finally, when the paw was affected, a maximum punctuation of 7.5 was directly attributed. Scores were added up and divided by the maximum necrotic tissue (5 nails + 5 fingers: 7.5) in order to get the percentage of necrotic tissue $\left(\frac{n^{\underline\circ}\;nailsx0.5+n^{\underline\circ}\;fingers\;x1}{7.5(maximum\;score)}\right)$.

### Tissue extraction

On day 21 after surgery, mice (n:32) were euthanized in a CO_2_ chamber. Left limbs muscles were harvested, and low frontal muscles (*tibialis anterior*) were fixed in 4% paraformaldehyde for 15 days prior cryoprotected in 30% sucrose in PBS 1X during 24 h. Tissue was then embedded in OCT and frozen for sectioning and immunohistochemistry (IHC) processing. Also, low back muscles (*gastrocnemius* and *soleus*) were snap-frozen in liquid N_2_ and stored at -80 ºC for further proteomic analysis.

### Immunohistochemistry analysis

OCT embedded tissues were cut transversally in 10 μm sections and placed in poly-L-lysine slides. In total, 3 tissue sections separated by 150 μm each were employed for vessel and cellular detection from all mice (n:8 per group), as described [5, 29, 30]. Primary antibodies employed were anti-α-actin smooth muscle (α-SMA) for vessel detection, anti-MOMA-2 for macrophages/monocytes detection, and anti-Ly-6G for neutrophils detection. Also, direct labeling with FITC-UEA1 was used to detect the presence of human endothelial cells (ECs), as described [29]. The entire tissue area was analyzed using Zeiss ApoTome.2, acquiring images at 20 × and visualizing with Zen 3.5 (Zeiss) software. Results were expressed as the number of blood vessels per mm^2^, number of cells per mm^2^ or blood vessels diameter (μm). Full information regarding antibodies can be found in Supplementary table S1.

### Sample preparation for MS analysis

Muscle samples from all groups (AT, CB, IC and Shams, n:8/group) were snap-frozen in liquid nitrogen prior to homogenization using a CP02 automated dry pulverizer (Covaris).

Homogenized tissue was further lysed in 1 ml of boiling buffer containing 5% SDS, 5 mM Tris (2-carboxyethyl) phosphine, 10 mM chloroacetamide, 100 mM Tris (pH 8,5), phosphatase inhibitors (1 mM NaF, 1 mM beta-glycerol phosphate and 5 mM of sodium orthovanadate) and protease inhibitors (Roche cOmplete™ Mini, EDTA free Protease Inhibitor Cocktail). Samples were denatured, reduced, and alkylated during 10 min at 95 ºC and 1400 rpm. Protein concentration was quantified through BCA method (according to manufacturer’s instructions, Pierce) and muscle samples were digested overnight using the protein aggregation capture (PAC) protocol implemented for the KingFisher Flex robot (Thermo Fisher Scientific) in 96-well format, as previously described [34, 35]. Briefly, the 96-well comb is stored in plate #1, and sample (3 mg) in plate #2 in a final concentration of 70% acetonitrile (ACN) and with magnetic Amine beads (Resyn Biosciences) in a protein/bead ratio of 1:2. Washing solutions are in plates #3–5 (95% ACN) and plates #6–7 (70% ethanol). Finally, plate #8 contains 300 µl of digestion solution composed of 50 mM ammonium bicarbonate, LysC (Wako, 1:250 enzyme:protein ratio) and Trypsin (Sigma Aldrich, 1:500 enzyme:protein ratio). PAC was performed in two steps of 1 mixing at medium speed, followed by 10 min pause each and sequential washes were carried out in 2.5 min and slow speed, without releasing the beads from the magnet. The digestion was set to 12 h at 37 ºC with slow speed. Protease activity was quenched by acidification to final 1% trifluoroacetic acid. Finally, we estimated a peptide recovery of 25%, and extracted the equivalent volume for 750 ng of peptide for LC–MS/MS analysis. Peptides were resuspended in 20 µl of 0.1% formic acid, were loaded directly into Evotips (Evosep) for full proteome analysis; and the remaining sample was loaded onto Sep-Pak cartridges (C18 1 cc Vac Cartridge, 50 mg – Waters).

### LC–MS/MS analysis

All samples were analyzed on the Evosep One system using an in-house packed 15 cm, 150 µm i.d. capillary column with 1.9 µm Reprosil-Pur C18 beads (Dr. Maisch, Ammerbuch, Germany), using the pre-programmed gradient for 30 samples per day. The column temperature was maintained at 60 ºC using an integrated column oven (PRSO-V1, Sonation, Biberach, Germany) and interfaced online with the Orbitrap Exploris 480 MS (Thermo Fisher Scientific, Bremen, Germany) using Xcalibur (tune version 1.1) and operating in independent data acquisition (DIA). Full MS resolution was set to 120,000 at m/z 200 and full MS AGC target was 300% with a maximum injection time (IT) of 45 ms. Scan mass range for full MS was set to 350–1400. AGC target value for fragment spectra was set at 1000%, and 49 windows of 13.7 m/z scanning from 361 to 1033 m/z were used with an overlap of 1 Da. Resolution was set to 15,000 and IT to 22 ms. Normalized collision energy was set at 27%.

### Raw data processing and protein identification

Raw files from muscle samples were processed using Spectronaut (v15.4) with a library-free approach (directDIA) employing mouse database (Uniprot reference proteome 2020 release, 21,989 entries). Moreover, carbamylation of cysteines was set as a fixed modification, while oxidation of methionines and acetylation of protein N-termini were set as possible variable modifications. Enzyme was established to Trypsin, data filtering to *q-*value and cross-run normalization was turned off. Rest of variables were set using default settings.

### Functional analysis

Functional analysis was done using Ingenuity® Pathway Analysis (IPA®, QIAGEN Redwood City, accessed in June 2023). The consideration of a statistically significant function was based on *p*-value < 0.05 and the z-score value.

### Western-blot analysis

In order to validate the proteomic results and evaluate possible ferroptosis in ischemic mice, the levels of the protein GPX4 were measured by western-blot (WB). Thus, tissue proteins (20 µg per sample) were loaded and separated on 12% acrylamide gels, and then transferred to PVDF membranes, which were next stained with Ponceau. Membranes were blocked with 5% milk 1 h at RT and immunoblotted with anti-GPX4 antibody (1:1000; ab125066) overnight at 4ºC. After several washes, membranes were incubated with anti-rabbit IgG secondary antibody HRP-conjugated (1:2500; 31460) for 1 h at room temperature. Chemiluminescent HRP detection substrate was used for image acquisition in a ChemiDoc Touch System (Biorad). Images from the Ponceau stained membrane were used for normalization as loading control (LC).

### Lipid peroxidation assay

Ferroptosis was also evaluated by measuring the levels of malondialdehyde (MDA) in the left limb adductors (n:8/group), using the Elabscience® Malondialdehyde (MDA) Colorimetric Assay Kit, TBA method. (E-BC-K025-S), following the manufacturer´s guidelines. Briefly, muscles were washed and lysated with PBS 1X, protein concentration measured with the BCA method, and incubated with the reagents for colorimetric assay. Absorbances were measured at 532 nm, and MDA concentration (nmol/mg prot) was calculated per sample group as indicated by manufacturers.

### Statistical analysis

The results regarding BF reperfusion, ischemic changes and vessel density analysis were evaluated with the GraphPad Prism 9 software (Boston, MA, USA). The normality of the variables was assayed by Shapiro–Wilk test, and a Kruskal–Wallis test and Dunn’s test as post hoc, or two-way ANOVA test with Tukey’s test for post hoc analyses were applied accordingly. Data were presented as mean ± SEM, and differences were considered statistically significant when *p*-value < 0.05.

Protein-related statistics were obtained with Perseus (v1.6.15.0) software [36]. Briefly, data were log2 transformed, 70% valid values were filtered, data were LOESS normalized and imputed by a constant value of 0.5 [37]. A two-sample student’s *T*-test was performed, and proteins were considered differentially expressed between the groups when *p*-value < 0.01. Protein changes were next confirmed with GraphPad Prism 9 software (Boston, MA, USA), and data were presented with box and plots graphs representing median, min and max value and showing all points. Also, receiver operating characteristic (ROC) curves were generated for some of the differentially expressed proteins (DEPs) by plotting sensitivity (%) against 100%—specificity (%), indicating the area under the curve (AUC) and 95% confidence intervals. Finally, the Spearman correlation test was performed to evaluate potential correlations between the intensity levels of some of the DEPs in the groups of interest.

## Results

### ECFCs + MSCs improve blood flow recovery in CLTI mice

A significant decrease of BF was seen in all mice right after FAL, compared with pre-surgical values and sham controls (≥ 80% reduction; Fig. 1B and C). In response to ischemia, a slight increase of BF (15.51%) was seen on day 7 in ischemic control mice (IC), treated with saline serum, compared to day 0 post-surgery values. By days 14 and 21, IC mice recovered up to 60% of BF ratios (left/right limbs), compared to normal, pre-ischemic condition (Fig. 1C). On the other hand, mice that received an intramuscular administration of either AT-ECFCs + AT-MSCs (AT) or CB-ECFC + AT-MSCs (CB), showed a significant recovery of BF already at day 7, detecting 70.31% (AT) and 63.58% (CB) of BF ratios in these mice (compared to baseline), which were significantly higher than IC group (AT, *p* < 0.0001; CB,* p* < 0.0015). Moreover, BF ratios were even higher on day 14 (AT, *p* < 0.0001; CB, *p* < 0.0001, *vs* IC) and day 21 (AT, *p* < 0.0008; CB, *p* < 0.0033, *vs* IC) in response to the cells, reaching more than 90% BF ratios in both cases (Fig. 1C). In terms of the cell source, the highest BF ratios were seen with the AT combination on days 7 and day 14, compared to the CB group, although by day 21 the recovery was similar with both sets of cells (91.16% and 91.63% BF ratios respectively; Fig. 1C).

### Co-administration of ECFCs and MSCs reduce necrotic progression in CLTI mice

Functional and ischemic symptoms (reduced motility, ulceration, necrosis) were evaluated along the assay, and images were taken for all mice on days 0, 1, 7, 14, and 21 (Supplementary Figure S2). After surgery (day 1), femoral ligated mice (IC, AT, CB) presented inflammation along the injured limb, some black nails, and even necrotic fingers in some of them, with significantly impaired motility, since most of them were not bearing properly on the injured toe or were limping in some cases (Supplementary Figure S3). Overall, the IC group showed the worst prognosis, with a drastic increase of necrosis already by day 7 (91.4% of the limb tissue affected), progressing up to (94.3% ± 15.7%) on day 21, with the highest percentage of necrotic tissue affected (Fig. 1D). Mice receiving cell therapy also presented ischemic symptoms along the 21 days, although the percentage of necrosis was lower already on day 7 (53.3% and 66.7% for AT and CB respectively), and the necrotic progression was slower in the treated mice than the IC group, with lower percentages on day 21 (82.5% and 73.3% for AT-ECFCs and CB-ECFCs respectively). Overall, the CB group had the slowest ischemic progression, with lower inflammation and lower necrotic rates along the assay (Fig. 1D). Similarly, according to mobility-related symptoms (Fig. 1E), the IC group showed the major impairment by day 7, compared to treated mice (AT and CB), being unable to completely recover or bear their weight by day 21. On the other hand, cell-treated mice progressively recovered their mobility ability, being nearly as motile as pre-surgery levels on day 21.

### ECFCs and MSCs promote collateral vessel formation and arteriogenesis in CLTI mice

We subsequently tested whether BF recovery was related to an increased vascularization. Overall, our results indicated that administration of AT promoted a significant increase in the number of vessels by day 21 (Fig. 1F), compared to SH (*p* < 0.019). The IC and CB groups also had higher number of vessels than the shams, although these differences were not statistically significant. Besides, further classification of vascular vessels per diameter size, indicated that CB-treated mice had more vessels with larger diameters (33.70% of vessel diameter ranged between 50 and 200 µm), compared to IC group (16.93%), AT group (19.39%) and SH group (8.42%) (Fig. 1G). Thus, while the AT combination promoted a higher increase of vessel density, with the CB cells the diameters of the newly formed vessels were wider. Representative IHC images of vessels detected within the tissues are shown in Fig. 1H.

### Proteome profile changes in response to ischemia and cell treatment at day 21

**Fig. 2:**
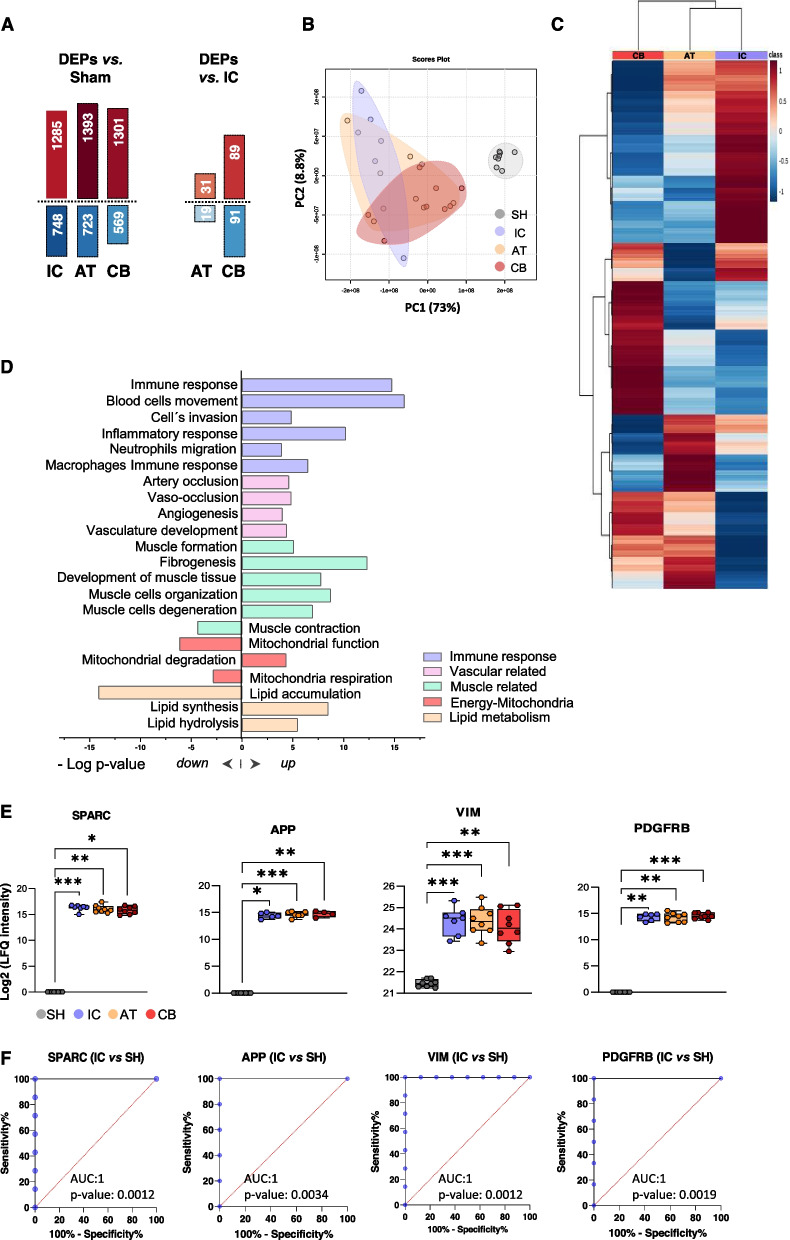
Proteomic changes in response to long-term ischemia.** A** Number of differential expressed proteins (DEPs) found up (red) and down-regulated (blue) in each group (IC, AT, CB) compared to Shams (SH), or in cell-treated mice (AT, CB) *vs* untreated ones (IC) (biological replicates, BR: SH, n:8; IC, n:8; AT, n:8; CB, n:8). **B** Principal component analysis (PCA) classification of all groups (BR: SH, n:8; IC, n:8; AT, n:8; CB, n:8). **C** Hierarchical clustering including the DEPs found in IC, AT, and CB compared to SH (*p*-value < 0.01), after T-test analysis (BR: SH, n:8; IC, n:8; AT, n:8; CB, n:8). **D** Graphical representation of the main enriched functions found up- or down-regulated in ligated mice *vs.* shams, based on the information provided by IPA (-log *p*-value) (BR: SH, n:8; IC, n:8; AT, n:8; CB, n:8). **E** Box and plots graphs representing the median, min and max values (with all points registered), of the label-free quantification (LFQ) intensities (Log2) registered for several proteins altered in ischemic groups (BR: IC, n:8; AT, n:8; CB, n:8) *vs* shams (BR, n:8). Statistical differences were found after performing Kruskal Wallis and post-hoc Dunn´s test, shown as as * *p*-value < 0.05, ** *p*-value < 0.01, *** *p*-value < 0.001, between BR: SH, n:8; IC, n:8; AT, n:8; CB, n:8). **F** receiver operating characteristic (ROC) analysis for these proteins in IC *vs* shams, with area under curve (AUC) and respective *p*-values

After mass-spectrometry analysis, a total of 4019 proteins were identified in all groups (Sham, IC, CB and AT). Further quantitative analysis reported on average 1326 ± 58 proteins up-regulated and 680 ± 96 down-regulated in all ischemic mice (IC, AT, CB), compared to shams, used as baseline control (Fig. 2A), while 217 proteins were up- or down-regulated between cell-treated (CB and/or AT) and IC mice (Fig. 2A). Finally, some DEPs were also identified between CB and AT mice (45 proteins up- and 42 down-regulated in CB *vs* AT). Full information regarding the identification and quantification data (intensity ratios and *p*-values) can be found in Supplementary Tables S3-S6.

The proteome profile of all ischemic mice was clearly differential when compared to shams, as observed by principal component analysis (PCA) (Fig. 2B). Among them, the profile of IC ischemic untreated mice was different than the cell treated groups (CB and AT), whose patterns did not entirely match between themselves, as corroborated by the hierarchical clustering analysis, having AT group an intermediate profile between CB and IC (Fig. 2C). These results suggest that most differential proteins were due to the ischemia but also as result of cell administration and in less extend, with the cellular type administered.

### Ischemia affects muscle integrity and vascular related proteins

**Fig. 3:**
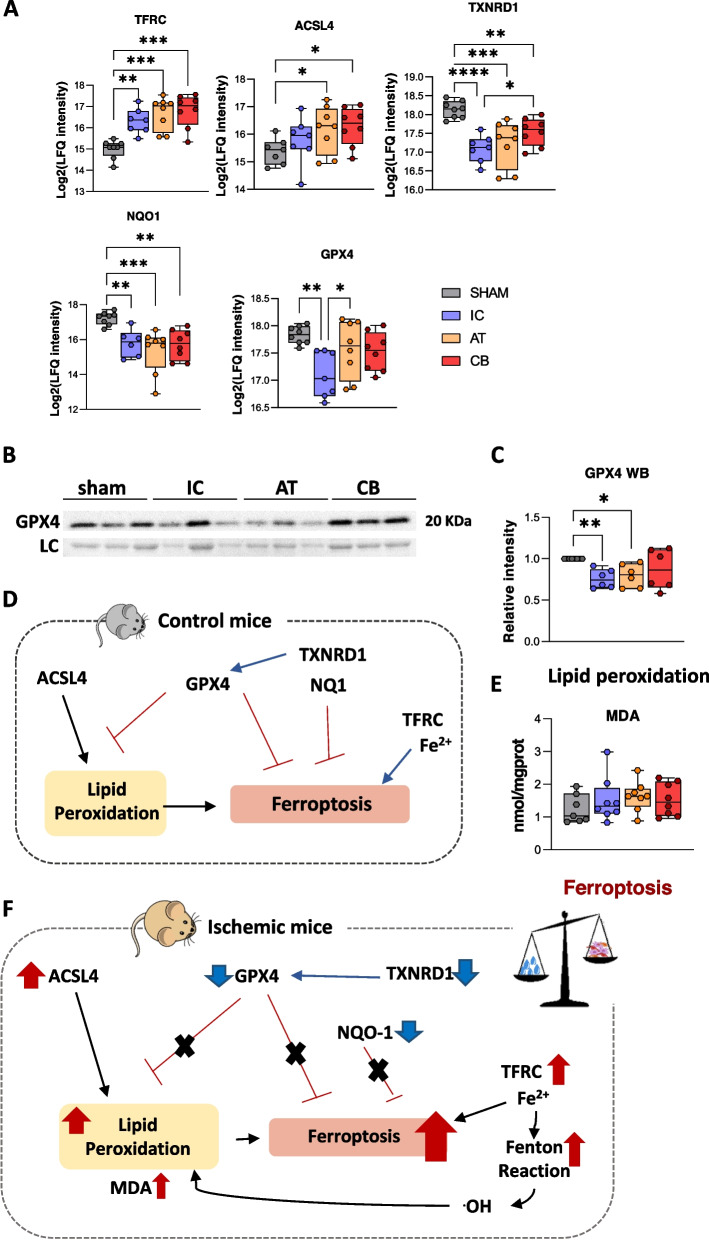
Ferroptosis upregulation in ischemic mice. **A** Proteomic analysis reported several proteins altered in FAL-ischemic mice (IC, untreated and AT or CB treated mice) compared to sham controls. A graphical representation of the changes seen for some of them is shown (representing the Log_2_ of the LFQ intensities registered by MS analysis). **B** A western-blot analysis was done to evaluated the expression changes for the protein GPX4 in the different groups (SH, IC, AT, CB, n:6 per condition). The image registered after staining the membrane with ponceau was used for normalization as loading control (LC). **C** Graphical representation of the expression changes detected by western-blot for GPX4, normalized versus shams intensities. **D** Graphical representation of some proteins identified associated to Ferroptosis. **E** The levels of Malondialdehyde (MDA) were also measured with a specific colorimetric assay, representing the concentration of MDA (mmol/mgprot) per group (n:8/group). Although no statistically significant differences were seen, the tendency indicated an increase of MDA in ischemic mice compared to shams. **F** Changes detected in ischemic mice representative of an upregulation of ferroptosis. Proteins included here: Transferrin receptor (TFRC); Thioredoxin reductase 1 (TXNRD1); Acyl-CoA synthetase long-chain family member 4 (ACSL4); Glutathione peroxidase 4 (GPX4); NAD(P)H quinone oxidoreductase 1 (NQO1)

According to functional enrichment analyses, the DEPs identified in ischemic mice after 21 days post-ligation correlated with an alteration (up or down-regulation) of a significant number of functions (Fig. 2D), including among others, an up-regulation of processes related to immune response, such as cell movement of blood cells (i.e. leukocytes, granulocytes, phagocytes…), invasion of cells, migration of neutrophils, as well as the immune response of macrophages (NCKAP1L, CD99, CAPG, CSF1R, LGALS3, ITGB2, C5, S100A8/A9 etc.). Also, several proteins altered in ischemic mice correlated with an up-regulation of vascular processes like artery occlusion or vaso-occlusion (PROC, PLG, PF4, etc.), while other proteins found up-regulated in ischemic mice (Fig. 2E and F) were related to the development of vasculature and angiogenesis (ABCC9, ANGPTL2, APP, LRP1, PDGFRB, SPARC, NPC2, HCK, VIM etc.…). Likewise, many proteins associated to muscle damage and dysfunction were identified (SPARC, LDB3, MYOZ1, GSN, etc.), detecting an up-regulation of muscle degeneration or necrosis of muscle cells, as well as proteins associated with muscle development (formation of muscle, organization of muscle cells, fibrogenesis, etc.) (ARRB2, CKM, CSPR3, TCAP, FN1, SERPIN1H, VAMP2, etc.). Other processes like apoptosis or ferroptosis were also found up-regulated in all ischemic mice, 21 days after FAL. In the case of ferroptosis, 41 DEPs were associated to this process, including proteins such as ACSL4 or TFRC (upregulated in ischemic mice), or GPX4 (down-regulated), known as markers of this pathway [38–41]. The down-regulation of GPX4 seen by proteomic analysis in response to ischemia was corroborated by WB. Also, an increase of MDA, a final product of lipid peroxidation, in ischemic mice (although not significant compared to shams), was detected. Both, GPX4 down-regulation and MDA increase, are closely linked to ferroptosis (Fig. 3).

These changes were accompanied by the alteration of energy production pathways, including the activation of mitochondrial dysfunction and degradation of this organelle (SOD1, SOD2, MFN1, ATG3, etc.), as well as dysregulation of the lipid metabolism, with an increase of lipids synthesis and hydrolysis, lower accumulation of lipids, and another lipid related functions (i.e. FABP5, FASN, HEXA, LIPE, PLIN1). Complete information including the functions altered in ischemic mice compared to shams can be found in Supplementary Table S7-S8.

### Cell therapy administration modulates the proteomic changes related to ischemia

**Fig. 4:**
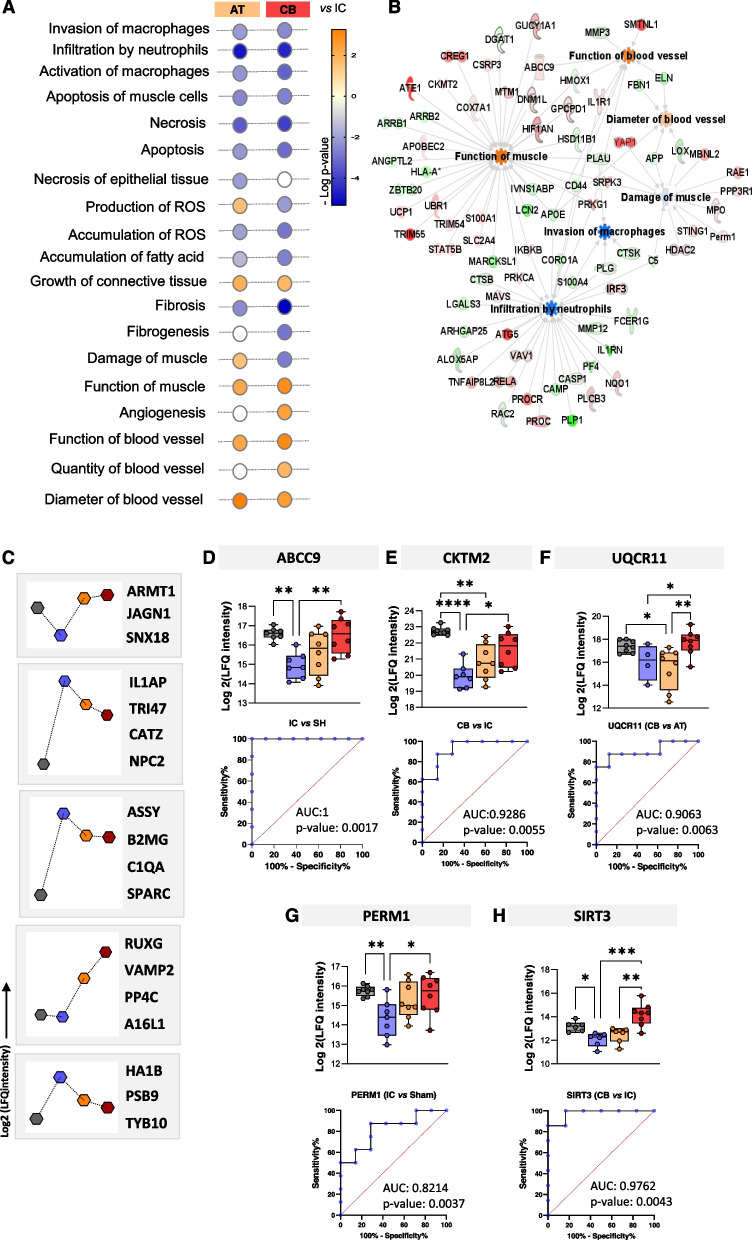
Proteomic changes related to cell administration. **A** Graphical representation of the main enriched functions found up- or down-regulated in cell treated mice (AT and CB *vs.* IC untreated ones (n:8 biological replicates, BR, per group), based on the information provided by IPA (-log *p*-value). **B** Ingenuity (IPA) functional network with proteins up- (red) or down-regulated (green) correlating with several functions altered in response to ischemia and modulated after cell administration (AT and CB *vs* IC, n:8 BR per group). **C** Representative graphs of some of the differential expression patterns most frequently seen (Log2 LFQ intensities), with the name of some proteins that follow such patterns (BR: SH, IC, AT, CB, n:8 per group). From **D** to **H** Box and plots graphs representing the median, min and max values (with all points registered) of the label-free quantification (LFQ) intensities (Log2) registered for several proteins with differential expression in AT or CB mice *vs* IC untreated ones, as well as the receiver operating characteristic (ROC) analysis registered for these proteins, indicating the area under curve (AUC) and respective *p*-values (BR: SH, IC, AT, CB, n:8 per group). Differences were considered significant when *p*-values < 0.05. *p-value < 0.05, *p-value < 0.01, *p-value < 0.001 after performing Kruskal Wallis and Dunn’s test as post-hoc

Many of the altered proteins in cell-treated mice, either the AT or CB combination, were also found in untreated ones, although these changes not always followed the same pattern seen for the IC group (as shown in Figs. 2C and 4A), detecting several functions dysregulated in response to ischemia that were modulated by the cells. Thus, according to IPA, several processes linked to immune response (immune cells mobilization and activation, invasion and activation of macrophages, or neutrophils infiltration), were downregulated in AT and CB *vs* IC mice, independently of the cell source (Fig. 4A and B). Similarly, the DEPs found in response to cell therapy, correlated with a decrease of cell death (apoptosis and necrosis) compared to IC, including a decrease of apoptosis of muscle cells in both cell-treated groups (STK4, YAP1, IL1RN, ATG7, etc.) (Fig. 4A).

In addition, we also found some differences between AT and CB which correlated for example with up-regulation of reactive oxygen species (ROS) production and lower down-regulation of muscle damage in AT mice compared to CB group (ej. PML, RELA, RIPK1…), although the accumulation of ROS diminished in both groups as well as others related with accumulation of fatty acids.

In terms of skeletal muscle integrity, some of the DEPs seen in AT/CB *vs* IC untreated mice were related to the enhancement of muscle function (ABCC9, ANGPTL2, DNM1L, CKM, etc.…) and the growth of connective tissue (Fig. 4B). Also, other processes such as fibrosis and fibrogenesis were down-regulated in both cell therapy groups (Acp5, AGK, ARRB2, ATG7, etc.), and again, this decrease was more pronounced in the CB group. In fact, functions related to muscle damage were reduced in CB mice although slightly up-regulated in AT, compared to IC untreated mice. For example, the levels of some of the proteins down-regulated after ischemia appeared to recover with the cell treatments (Fig. 4C). Moreover, proteins like ABCC9, CKMT2, UQCR11, PERM1 or SIRT3 were highly discriminating between IC and SH or moreover, between cell treated or untreated mice, with high AUC values in the ROC curves (Fig. 4D-H). For instance, ABCC9 (r: -0865, *p*-value:0.026) and CKTM2 (r: -0.786, *p*-value: 0.048), they also showed strong negative correlations between their expression levels in IC *vs* shams or CB *vs* IC mice respectively. Lastly, an enhancement of proteins associated to angiogenesis (AKAP1, APP, BIRC6, EMILIN1, FBLN2, etc.), vessel density or arteriogenesis, was seen in the CB and AT mice compared to IC (APP, ELN, FBN1, LOX) (Fig. 4A). Full information including the functions altered in cell treated mice compared to ischemia control can be found in Supplementary Table S9.

### ECFCs and MSCs administration diminished immune cells recruitment to the injured area

**Fig. 5:**
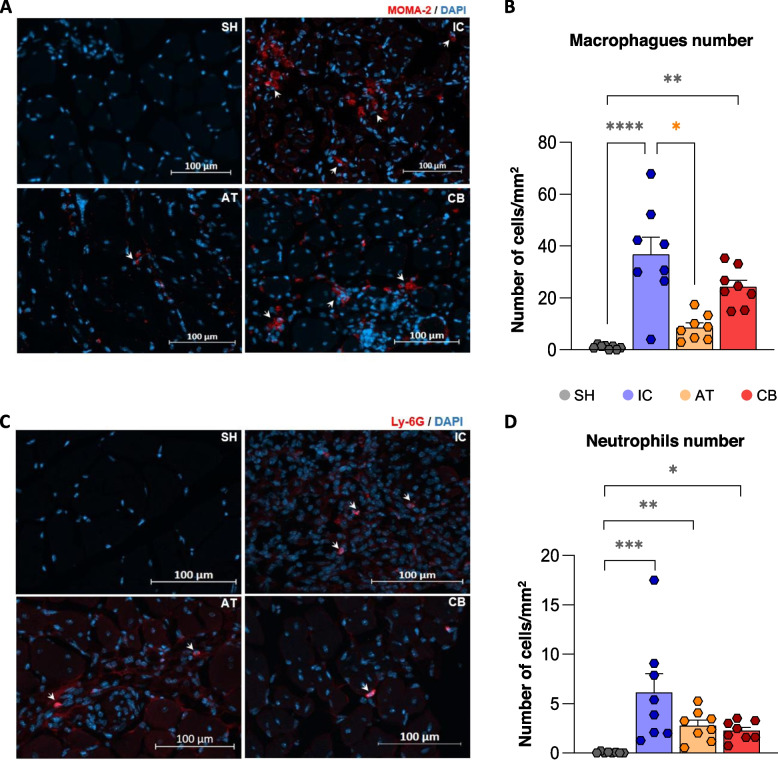
Cell administration modulates the recruitment of immune cells into the ischemic tissues. **A** Representative images of macrophages (MOMA-2, red) and **B**) neutrophils (Ly-6G, red) detected on day 21 by IHC in the limb muscles of all groups (BR: SH, IC, AT, CB, n:8 per group; technical replicates: n:3/group). Graphical representations of the number of **C**) macrophages and **D**) neutrophils quantified (cells/cm^2^) in IHC sections of all groups (BR: SH, IC, AT, CB, n:8 per group; technical replicates: n:3/group). Data are presented as the mean ± SEM, and statistically significant differences are shown after performing Kruskal–Wallis and Dunn’s test as post-hoc (* *p*-value < 0.05, ** *p*-value < 0.01, *** *p*-value < 0.001)

Although we could not find traces of human cells after 21 days post-administration (Supplementary figure S4), we observed a higher number of macrophages (*p* < 0.0001) and neutrophils (*p* < 0.0001) in the ischemic muscle of IC group after 21 days of FAL, compared to shams, where both immune cells were barely detected (Fig. 5A-D). The quantity of macrophages was lower in AT group when comparing with IC mice (*p*: 0.050) and CB group (*p*: 0.001, compared to shams). In the case of neutrophils, a low number was found in CB mice and AT mice, whereas significantly higher than shams (CB, *p*: 0.030; AT, *p*: 0.007).

## Discussion

Cell therapy is being widely tested as a treatment for “no-option” CLTI patients in both pre-clinical and clinical stages [8, 42–45]. However, the discrepancies registered in these studies as result of differences in cell types employed, cell combination, administration routes or doses applied, make necessary to reach a consensus regarding the optimal “cellular-cocktail” for these patients, and set-up optimized but well stablished protocols prior further application into the clinic [8, 46, 47].

An important factor to address in cell therapy is the choice of cell source. In this sense, although the therapeutic potential of ECFCs and MSCs has been extensively investigated in the context of vascular regeneration [48, 49], all studies have always used ECFCs derived from umbilical cord blood (CB) or peripheral blood (PB). CB-ECFCs have proven to show great angiogenic potential, but their use still has some ethical issues due to their embryonic origin. On the other hand, PB-derived ECFCs can be obtained from adults, but they are extremely rare in adult peripheral blood [22, 50, 51]. Consequently, the success of their extraction is highly variable, making their extraction unreliable across patient populations. Alternatively, the isolation of ECFCs from AT is highly efficient [22], providing an alternative that is easily achievable, and susceptible to be applied as allogenic or autologous therapy. Finally, AT is also chosen as an optimal source of MSCs, with easier and less invasive access than the BM itself. Besides, recent studies have shown a better recovery of HLI animal models treated with AT-MSCs than with BM-MSCs [52].

In the current study we have evaluated, for the first time to our knowledge, the regenerative potential of a combination of adipose tissue (AT)-derived cells, AT-ECFCs and AT-MSCs, in a murine model of CLTI, compared to the effect of CB-ECFCs with AT-MSCs. Moreover, the molecular mechanisms altered in CLTI and in response to both combinations have been evaluated at a proteomic level.

Based on our results, FAL significantly reduced BF (> 80%), which was accompanied by inflammation, gradual presence of black nails and necrotic fingers, as well as a negative impact in the animals’ motility. Despite this, a recovery in BF could be seen by day 7, even in IC untreated mice, which might be explained by repairing mechanisms triggered in response to hypoxia an ischemic events, such as the formation of new capillaries and new muscle fibers [53], in an attempt to recover from the absence of oxygen and lack of nutrients, as previously reported [54]. Such vessel sprouting takes place during the first days post-ischemia, even in non-treated tissues [30]. Afterwards, the newly formed vessels either grow and become functional or, on the contrary, they are immature and fragile, and they might contribute to the tissue instability [55, 56]. Remarkably, by day 21, the BF recovery seen after cell administration, for instance co-administration of AT-MSCs and ECFCs (either CB or AT source), was higher than in IC untreated mice, where perfusion ratios were significantly lower, in agreement with previous studies [49, 57]. Besides, although the highest BF ratios were observed with the AT combination on days 7 and 14, compared to the CB group, by day 21 the recovery was similar with both sets of cells. Furthermore, our data indicated that the transplantation of AT-ECFCs seemed to foster BF reperfusion by promoting a higher number of functional vessels, while in CB-ECFCs treated mice, such improvement appeared associated to an increase of lumen vessel diameters, as further corroborated by proteomic results. The fact that we did not detect any traces of human cells after 21 days post-injection, suggests that ECFCs and MSCs might exert their long-term angiogenic effect in a paracrine fashion, in agreement with previous findings [5, 21, 30, 58–60], with many studies reporting the presence of these cells in the ischemic muscles up to 14 days [57, 61].

Overall, the LFQ proteomic analysis corroborated previous results from our lab regarding the long-term effect of the ischemic process [30], with an important number or protein changes detected in the ischemic limbs that reflected an enhanced immune and inflammatory response, as well as changes associated to vascular-related processes and muscle damage itself, which was accompanied, among others, by an alteration of energy homeostasis, oxidative stress, mitochondrial dysfunction or dysregulation of lipid metabolism, as further disclosed above.

### Proteomic changes related to vascular damage in ischemic mice

Based on functional classification analyses, the differential protein profiles identified represented an up-regulation of vessel occlusion, as expected, as well as proteins associated with an activation of angiogenesis and vasculature development. Among the proteins up-regulated in all ischemic mice (IC, CB and AT) we found Angiopoietin-like Protein 2 (ANGPTL2), a well-known pro-angiogenic agent that appears to enhance ECFCs vasculogenesis in vitro [62] and whose expression has been associated with increased number of blood vessels in mice [63, 64]. Similarly, up-regulation of the Low-density lipoprotein (LDL) receptor-related protein-1 (LRP1), might be part of a repairing mechanism activated in ischemic tissues, given the role of LRP1 in the regulation of several signaling pathways responsible for maintaining and restoring vascular homeostasis [65]. Likewise, the Amyloid precursor protein (APP), associated to amyloid plaque formation and Alzheimer, has been found to promote angiogenesis, stimulating ECs migration, proliferation and formation of new vessels, at least in vitro [66]. Besides, the Platelet-derived growth factor receptor beta (PDGFRB), was also found up-regulated in ischemic mice. This pro-angiogenic factor is secreted from the endothelium of angiogenic sprouts, attracting comigration pericytes [67–69], and it also stimulates vascular smooth muscle cells proliferation and induces mural cell fate in MSCs [23, 69, 70].

Some of the proteins found in ischemic mice, however, were differentially expressed in cell-treated mice compared to untreated ones, correlating with a higher activation of angiogenic processes in CB or AT mice than in IC 21 days post-FAL. Among them, the Adenosine triphosphate (ATP)-binding cassette (ABC) transporter ABCC9, also known as sulfonylurea receptor 2 (SUR2), is a component of the ATP-sensitive potassium (K_ATP_) channel responsible for ATP-dependent inward K^+^ transport [71]. K_ATP_ channels have been identified as important mediators of ischemic preconditioning, limiting ischemic damage [72], and they also constitute key modulators of vascular smooth muscle activity by regulating blood pressure and episodic coronary artery tone [73]. Remarkably, ABCC9 was down-regulated in IC untreated mice, while its levels were restored with CB and more significantly with AT cells. This might represent an alteration of the K + transport through K_ATP_ channels after ischemia which was somehow restored in response to cell treatment, and for instance, a better recovery of the vascular tone as well in these mice.

### Immune response and altered metabolism in ischemic mice

In terms of inflammation, numerous proteins were found up-regulated in ischemic mice compared to shams. For example, the complement C5 protein has been widely associated to ischemia reperfusion injury or activation of neutrophils [74–76], as well as with the progression of atherosclerotic lesions [77]. Similarly, Galectin-3 (LGALS3) participates in the modulation of the inflammatory response as an important mediator of the reparative process driven by macrophages, and it has been associated with ischemia injury progression as well [78, 79]. Finally, other proteins up-regulated in ischemic mice were the S100A8/S100A9 complex, an important component in neutrophil mobilization [30, 80], or the transmembrane protein beta-2 integrin (ITGB2), which is expressed exclusively in leukocytes after an activation signal, promoting neutrophil adhesion strengthening, cell spreading and crawling [81–83]. The effects from the femoral blockade and hypoxic conditions were also reflected by alteration of the glucose metabolism (i.e. ALDOA, GAPDHS, PFKP, PGAM2, SDHB), with many pathways related that appeared down-regulated even at the long-term (glycolysis, gluconeogenesis and oxidative phosphorylation steps). All these processes are required to maintain energy balance and normal physiological function [84, 85]. Similarly, energy imbalance was represented by down-regulation of proteins affecting mitochondrial function integrity and mitochondrial respiration (TFAM, SOD1, SOD2, MFN1, BCS1L, UQCR11) [86–88], as well as proteins related to fusion/fission mitochondria processes and mitophagy (SEPTIN2, OPA1, BAX, BAK1, ATG3, AIFM1), while there was an up-regulation of proteins associated to mitochondrial degradation (ADH5, ADIPOQ, AGT, AIFM1, APP, ATG3, BAK1, BAX, DNM1L, etc.) [89–93]. On the other hand, glucose imbalance was accompanied by up-regulation of lipid metabolism, both lipid synthesis and lipid hydrolysis (FABP5, FASN, HEXA, LIPE, PLIN1, etc.). Cell administration, however, reverted the levels of some energy related proteins down-regulated in IC. For instance, proteins like PGC-1/ERR-induced regulator in muscle 1 (PERM1), were up-regulated in cell-treated mice compared to IC. PERM1 is a striated muscle-specific regulator of mitochondrial bioenergetics, required for the high ATP demanding contractile activity of muscle [94]. Also, in cardiomyocytes, PERM1 protects against stress-induced cellular damage because of hypoxia [95]. Other proteins like the Ubiquinol-cytochrome c reductase (UQCR11) decreased significantly in IC and AT mice, but their levels recovered in the presence of CB combination. UQCR11 is a core subunit of mitochondrial complex III, an essential component of the mitochondrial electron transport chain. Thus, although further studies should validate these results, at least in this case CB appeared to be beneficial to restore mitochondrial function and therefore to promote energy restoration in the ischemic muscles.

### Modulation of necrotic processes in cell-treated mice

Other common pathways associated to ischemia such as apoptosis, necrosis and cell death were found up-regulated in ischemic mice (both, IC untreated and AT or CB treated ones) although, based in our data, cellular administration somehow modulated and reduced the necrotic process, compared to IC untreated mice. Indeed, according to the necrotic scores applied, IC mice presented the highest percentage of necrotic tissue along the assay, while the necrotic ratios in CB or AT mice, although high, were slowed down, mainly in the presence of CB-ECFCs. Moreover, the CB group had the slowest ischemic progression along the assay. Interestingly, together with necrosis and apoptosis, a significant upregulation of ferroptosis, an important regulatory mechanism that induces skeletal muscle cell death and prevents skeletal muscle proliferation and differentiation [40], was also reported in all ischemic mice. Indeed, the changes seen for several proteins related to this process (ACSL4, TFRS, GPX4, etc.) [38, 39, 41], as well as the increase seen for the final product of lipid peroxidation, MDA [96], were indicative of ferroptosis up-regulation after 21 days of FAL induced ischemia. These results indicated that cell administration, at least in the case of ferroptosis, was not sufficient to restrain this process.

### Ameliorated immune response in cell treated mice

Similarly, despite the up-regulation seen of immune related pathways in response to ischemia, one of the main achievements of cell administration was, according to the proteomic analysis, a general down-regulation of inflammatory and immune response processes in AT/CB treated mice compared to IC untreated ones. These results were validated by IHC, detecting a higher number of macrophages and neutrophils in IC ischemic muscles 21 days post-FAL, compared to non-ischemic shams, but also to cell-treated mice. These results agreed with previous findings from our lab after the administration of CACs [30], and moreover, with studies reporting that a combination of ECFCs and MSCs from adipose tissue modulated and reduced neutrophils recruitment and activation in grafts implanted into mice as the vasculature matured [97]. Remarkably, the AT group presented the lower number of both, macrophages and neutrophils, in the injured tissue. Thus, a decrease in macrophage activation, neutrophil trafficking, adhesion capacity and chemotaxis was observed, with proteins down-regulated (ALOX5AP, CD14, CORO1A, CTSB, FCER1G, IL1RN, LBP) [98–105] and up-regulated (NQO1, PROCR, TIPE2) in AT and CB mice compared to IC ones, as it was also previously reported [106–108]. Overall, our results indicated that the combination of AT-MSC with either AT- or CB-ECFCs alleviated the impact of the negative effects of ischemia, reducing inflammation but also tissue necrosis.

### Muscle regeneration and tissue repair

In terms of muscle damage and tissue repair, many proteins associated either with muscle degradation, fibrosis, or necrosis of the skeletal muscle itself were identified, but also proteins involved in muscle regeneration. In this regard, based on the proteomic results, the administration of cells reinforced the repairing processes, with down-regulation of fibrosis and muscle damage (more intense in CB treated mice than AT ones) compared to IC, and up-regulation of proteins that participate in the reorganization and remodeling of muscle tissue fibers.

Among the DEPs identified, a critical protein found was Cysteine and Glycine Rich Protein 3 (CSRP3, also known as muscle LIM protein or MLP), which participates in muscle differentiation and maintenance of the contractile apparatus. Moreover, CSRP3 might activate autophagy in response to acute starvation and hypoxia. Autophagy is required to preserve the myotube structure and function during myotube formation and thereby prevents alteration of myotube morphology or integrity [109]. Therefore, the up-regulation of CSRP3 in AT and CB mice compared to IC might represent a positive effect of these cells, first to prevent the accumulation of damaged materials inside the cells, and second to promote muscle differentiation and repair.

Vesicle-associated membrane protein 2 (VAMP2, also known as synaptobrevin-2), which was found up-regulated only in response to cell treatment (AT and CB *vs* SH and IC), is involved in membrane trafficking, with a potential role in muscle regeneration [110, 111]. Initially identified in synaptic vesicles from rat brain [112], VAMP2 has been also detected in quiescent muscle satellite cells. Moreover, VAMP2 has been found upregulated in immature myofibers during muscle regeneration, in correlation with the release of VEGF, IGF and other growth factors by these cells.

Another important protein required to ensure proper functioning of the muscles is creatine kinase (CKM), an enzyme that catalyzes the reversible transfer of γ-phosphate from ATP to creatinine to produce phospho-creatine (PCr) and ADP, ensuring an efficient cytosolic storage of high-energy phosphates for rapid, focal ATP replenishment. The Cr/CK system provides an indispensable support to maintain cellular homeostasis when imbalances in ATP supply and demand [113]. In response to ischemia, a significant down-regulation of CKM was seen in our mice even 21 days post-FAL, what is associated to down-regulation of PCr as well as ATP levels, and reduction of energy availability, essential for the correct functioning of the muscle. Remarkably, although CKM levels were still lower in cell treated mice than control shams, we could see in AT and more significantly CB treated mice, a significant recovery of CKM levels compared to IC, which might have also contributed to a better recovery of ischemic muscles by a higher availability of energy resources.

### CB vs AT response

Finally, in terms of the cell combinations applied, either CB- or AT-ECFCs and AT-MSCs, both seemed equally efficient in promoting revascularization of the ischemic tissues, since they both achieved a significant perfusion recovery at the long-term, with modulation of the inflammatory process and reduction of the necrotic progression, compared to IC untreated mice. Nevertheless, while BF recovery seemed initially faster with the AT combination, probably because these cells induced angiogenesis and formation of a higher number of vessels, the recovery with CB was similar after 21 days, enhancing reperfusion by the development of wider vessels. Besides, the CB group had the slowest ischemic progression along the assay. In terms of the molecular mechanisms altered in response to ischemia, slight differences were seen between AT and CB treated mice. Several proteins had different tendencies in these groups and even presented statistically significant up- or down-regulation between them, although the overall response was similar. Nevertheless, in the AT group there was up-regulation of ROS production, as well as slightly higher muscle damage compared to CB, where fibrosis was also less pronounced, according to our functional analysis, than in AT treated mice. For example, the mitochondrial protein NAD-dependent deacetylase sirtuin-3 (SIRT3) was down-regulated in IC and AT mice compared to Shams, but its levels recovered in CB mice. Remarkably, SIRT3 is known to protect against ROS-induced damage by reducing ROS and influences many energy metabolism processes through deacetylation of key enzymes [114].

## Conclusions

Overall, we have demonstrated the potential of using a cellular combination with AT-derived cells, AT-ECFCs and AT-MSCs, to promote revascularization in a murine model of CLTI, with similar effects to those seen with the cocktail CB-ECFCs and AT-MSCs. Both combinations promoted a significant recovery of blood flow and a modulation of the inflammatory and necrotic processes, together with a reduction of muscle damage. Moreover, we have provided here, to the first time to our knowledge, a wide view of the molecular mechanisms altered in CLTI muscles, and many proteins involved in the repairing mechanisms promoted by cell treatments have been identified by application of high-throughput quantitative proteomics.

Regarding the cell source, the combination of CB-ECFCs and AT-MSCs seemed more efficient in reducing the necrotic process, and it could promote a better recovery of the ischemic tissue than the AT combination, although both cellular cocktails promoted similar reperfusion ratios in CLTI mice at the long-term. Thus, although the application of the CB combination seems initially a better approach, its use might depend, at the end, on the availability of these cells, and on the regulatory and ethical issues associated to embryonic cells. Alternatively, adult AT-ECFCs, from the same source than the AT-MSCs, are easier to get and could be applied within an autologous therapy. On the other hand, proteomic data suggested that using adult AT-ECFCs might imply the risk to achieve lower muscle and tissue restoring. Moreover, our results were obtained with cells from healthy donors. Therefore, further studies should evaluate the effect of AT-cells from CLTI patients, since their regenerative and angiogenic potential of ECFCs might be impaired. Nevertheless, despite these limitations, our results corroborate the potential use of a combination of MSCs and ECFCs, whether from CB or AT origin, as an optimal approach to enhance revascularization in CLTI, opening the possibility to apply autologous therapies to these patients with adult-derived cells.

### Supplementary Information

Supplementary Materials: Figure S1: Representative images of the femoral artery ligation (FAL) performed as well as the injection sites for cell administration; Figure S2: Evaluation of the progression of ischemic symptoms within time; Figure S3: Evaluation of the ischemic symptoms within time; Figure S4: Representative images of ECFCs detection through IHC.

Supplementary Materials: Table S1: Primary and secondary antibodies employed in this study; Table S2: Tarlov score, ischemia score and modified ischemia score; Table S3: Normalized and log-2 transformed protein intensities across all samples; Table S4: Differentially expressed proteins (DEPs) between Ischemia *vs.* Sham; Table S5: DEPs between AT/CB* vs.* Ischemia control; Table S6: DEPs between CB* vs.* AT; Table S7: Functional classification of DEPs in response to ischemia; Table S8: Functional classification of canonical pathways in response to ischemia; Table S9: Functional classification of DEPs in response to cell treatment.

## Data Availability

The data underlying this article are available in the article and in its online supplementary material. In addition, mass spectrometry proteomics data have been deposited to the ProteomeXchange Consortium via the PRIDE [115] partner repository with the data set identifier PXD046843.

## Abbreviations

ACN: Acetonitrile
αSMA: Alpha-smooth muscle actin
AT-ECFCs: Adipose tissue derived-Endothelial Colony-Forming Cells
AT-MSC: Adipose tissue-derived mesenchymal stem cells
BF: Blood flow
CB-ECFCs: Umbilical Cord Blood-derived Endothelial Colony-Forming Cells
CLTI: Critical Limb-Threatening Ischemia
HLI: Hind limb ischemia
MDA: Malondialdehyde
MSCs: Mesenchymal Stem Cells
PCA: Principal component analysis
