# Supplementary material for: Unraveling the differential mechanisms of revascularization promoted by MSCs & ECFCs from adipose tissue or umbilical cord in a murine model of critical limb-threatening ischemia
Source: J Biomed Sci. 2024 Jul 15;31:71. doi: 10.1186/s12929-024-01059-w (PMC11247736; doi:10.1186/s12929-024-01059-w)
Supplement: Supplementary file 2 — Supplementary Materials: Table S1: Primary and secondary antibodies employed in this study; Table S2: Tarlov score, ischemia score and modified ischemia score; Table S3: Normalized and log-2 transformed protein intensities across all samples; Table S4: Differentially expressed proteins (DEPs) between Ischemia vs. Sham; Table S5: DEPs between AT/CB vs. Ischemia control; Table S6: DEPs between CB vs. AT; Table S7: Functional classification of DEPs in response to ischemia; Table S8: Functional classification of canonical pathways in response to ischemia; Table S9: Functional classification of DEPs in response to cell treatment. [file 12929_2024_1059_MOESM2_ESM.docx]

**Supplementary Materials of “Unraveling the differential mechanisms of revascularization promoted by MSCs & ECFCs from adipose tissue or umbilical cord in a murine model of critical limb-threatening ischemia”**

**Full description of the femoral artery ligation performed.** Balb-c nude mice (n:24) were anesthetized with ketamine (100 mg/kg) and xylazine (10 mg/kg) by subcutaneous administration, and then placed in supine position over a heated pat to proceed with surgery. After extending and securing both limbs with a piece of tape, the skin of the left limb was wiped with betadine, and an incision of the left limb skin of approximately 1 cm long was carried out with fine forceps and surgical scissors from the knee towards the medial thigh, and the subcutaneous fat tissue surrounding the muscle was carefully removed using cotton swabs with PBS. A dissection microscope at 10-20X magnification was used to obtain a better image of the hind limb region.

Next, an incision was carried out to dissect the subcutaneous fat tissue in order to reveal the left femoral artery, and a retractor was used to open the wound and left the artery uncovered for ligation. Carefully, the femoral vein and nerve were separated from the femoral artery with fine forceps, in order to pass a strand of non-absorbable 6/0 silk suture (Silkam, C0765066) underneath the proximal site of the artery, occluding the artery with double knots. The same procedure was then done at the distal side of the artery to apply a second occlusion in this side. Once the FAL was carried out, the retractor was removed and the incision was closed the incision using 5-0 Vicryl absorbable sutures. Once the incision is closed, animals were placed on top of a second heated pad to proceed with blood flow measurement under the Periflux camera (Perimed, 2-3 min maximum), and finally we leave the mice in the recovery cage and monitor continuously until awake. After that, mice received analgesic injected intraperitoneally (Ketoprofen, 2 mg/kg) for three consecutive days.

Alternatively, sham mice (SH, n:8) underwent all the steps described, including the removal of the fat covering the femoral artery but closing right afterwards the incision, without performing any ligation or occlusion. These mice were employed as a surgery control for vascular density changes, immune cell detection and proteomic assays.

Twenty-four hours after surgery, mice received 3-4 injections of either 50 μl of saline serum (IC: Ischemic control mice) or 50 μl of cells in saline serum (either AT and CB combination), in different sites of the muscles surrounding the femoral artery: low frontal muscles (tibialis anterior), low back muscles (gastronecmius) and middle muscles (biceps femoris), as indicated in materials and methods.

**Supplementary Figure S1**


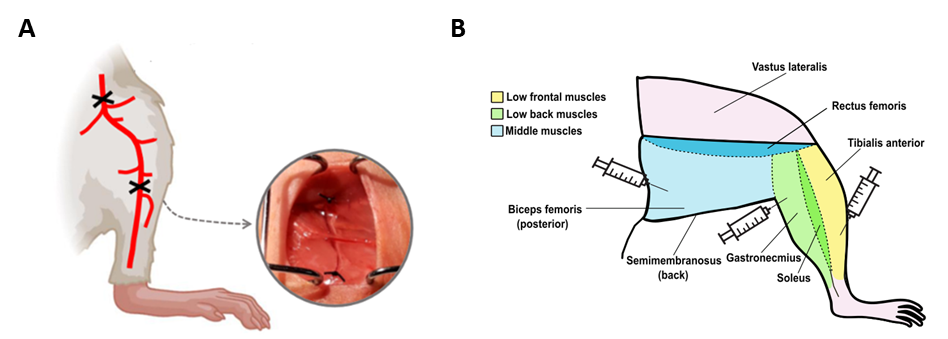


*Image modified from Beltran-Camacho L et al[1].

**Figure S1. Representative images of the femoral artery ligation (FAL) performed as well as the injection sites for cell administration**. A) FAL was carried out in the left limb of Balb-C nude mice (n:24), applying double knots in the distal and proximal sides of the femoral artery. B) In total, 50 ml of saline serum (IC. Ischemic control, untreated mice) or 50 μl of cells in saline serum (either AT or CB cells), were injected in different sites of the muscles surrounding the femoral artery, 24 hours post-FAL surgery, as indicated in the figure.

**Supplementary Figure S2**


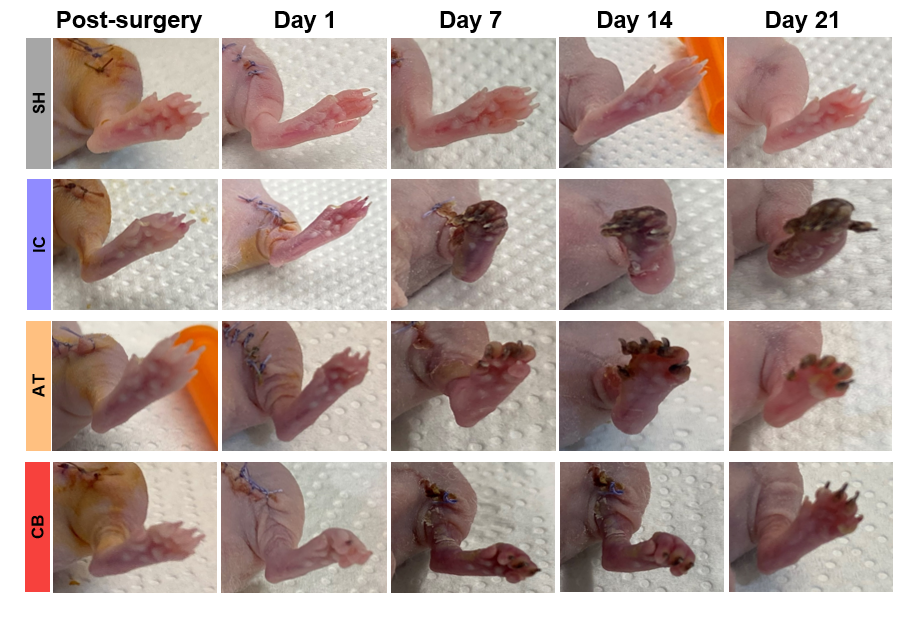


**Figure S2. Evaluation of the progression of ischemic symptoms within time.** (**A**) Representative images of the evolution of ischemic symptoms in mice post-surgery and on days 1, 7, 14 and, 21.

**Supplementary Figure S3**

**
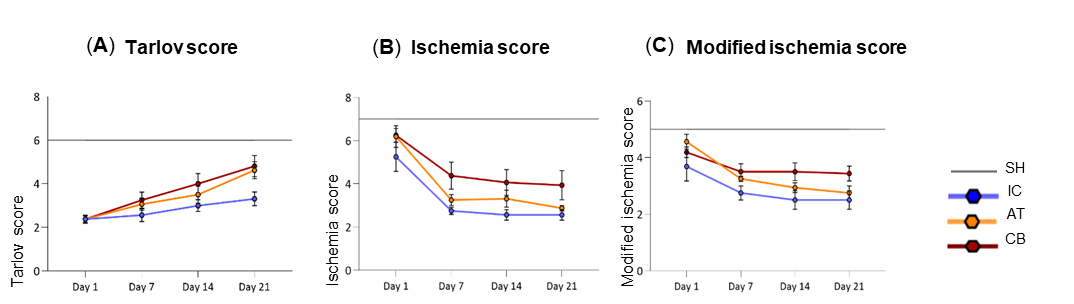
**

**Figure S3. Evaluation of the ischemic symptoms within time.** Graphical representation of Ischemic changes according to (**A**) Tarlov score, (**B**) Ischemia scores, (**C**) Modified ischemia scores, described in supplementary Table S2.

**Supplementary Figure S4**


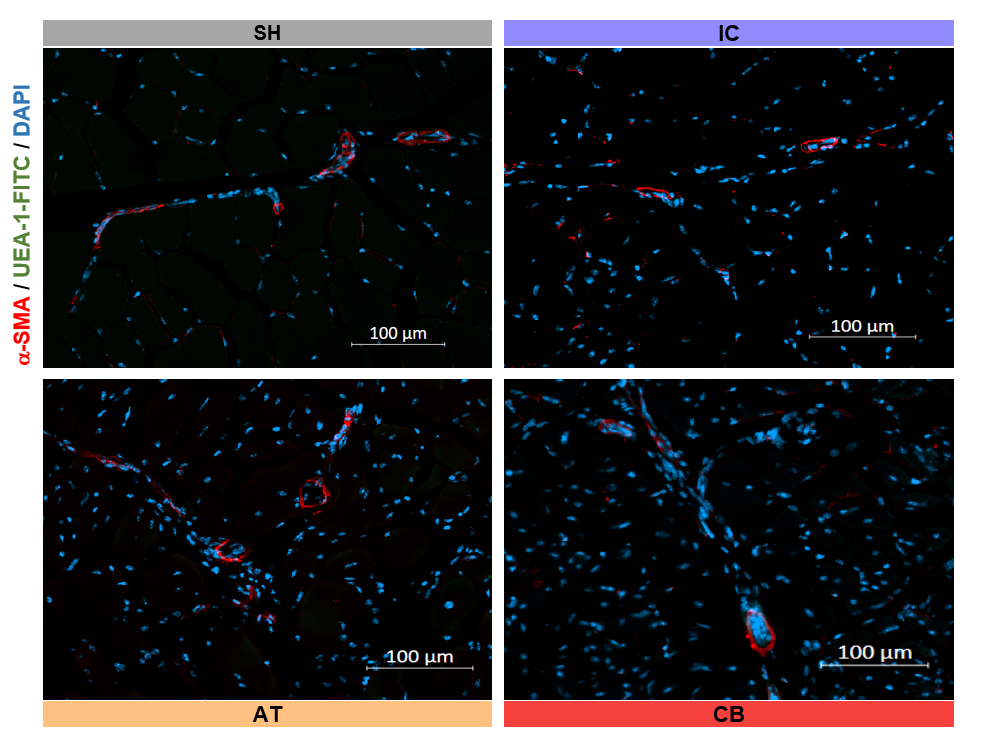


**Figure S4. Representative images of ECFCs detection through IHC.** Images after performing IHC analysis from low frontal muscles at day 21 using UEA-1 FITC (green).

**Supplementary Table S1. Primary and secondary antibodies employed in this study.**

| **Antibody** | **Reactivity** | **Dilution** | **Supplier** | **Reference** | **Lot #** | **Used in** |
| --- | --- | --- | --- | --- | --- | --- |
| CD14-PB | H | 1:25 | Biolegend | #367121 | B271628 | FC |
| CD31-FITC | H | 1:25 | Biolegend | #303103 | B224876 | FC |
| CD34-APC | H | 1:25 | Biolegend | #343607 | B223919 | FC |
| CD45-PB | H | 1:25 | Biolegend | #368539 | B264395 | FC |
| CD73-FITC | H | 1:25 | Biolegend | #344015 | B224217 | FC |
| CD90 (Thy1)-PE | H | 1:25 | Biolegend | #328109 |  | FC |
| CD133-PE | H | 1:25 | Miltenyi Biotec | 130-098-826 | 5140404153 | FC |
| CD146-PE | H | 1:25 | Biolegend | #361005 | B264161 | FC |
| CD309-PE | H | 1:25 | Biolegend | #359903 | B245460 | FC |
| IgG1 isoptype | H | 1:25 | Becton-Dickinson | 345816 |  | FC |
| CD3-PE | H | 1:25 | Biolegend | #317308 |  | FC |
| UEA-1-FITC | H | 3:1000 | Sigma-Aldrich | L9006 | 072M4026V | IHC |
| a-smooth muscle actin | M | 1:500 | Sigma | A5228 | 029M4807V | IHC |
| MOMA-2 | M | 1:500 | Sigma-Aldrich | MAB1852 | 3026751 | IHC |
| Ly-6G | M | 1:500 | Biolegend | 127601 | B265458 | IHC |
| Alexa Fluor 555 | M | 1:500 | Thermo Fisher | A21422 | 1837985 | IHC |
| Alexa Fluor 555 | R | 1:500 | Thermo Fisher | A21434 | 1907302 | IHC |

*H: Human; M: Mouse; R: Rat; FC: Flow cytometry; IHC: Immuhistochemistry.

**Supplementary Table S2.** (**A**)  **Tarlov score[2, 3].** Functional scoring evaluating motility, leg movement and difficulty walking. (**B**) **Ischemia score[4]**. Functional scoring to evaluated ischemic symptoms, advance of the characteristics along the leg. (**C**) **Modified ischemia score[4].** Functional scoring to evaluated ischemic symptoms, modified to be more correctly for mice.

**A)**

| **Tarlov score** |  |
| --- | --- |
| 0 | No movement |
| 1 | Barely perceptible movement, non-weight bearing |
| 2 | Frequent movement, non-weight bearing |
| 3 | Support weight, partial weight beaing |
| 4 | Walks with mild deficit |
| 5 | Normal but slow waking |
| 6 | Full and fast walking |

**B)**

| **Ischemia score** |  |
| --- | --- |
| 0 | Auto-amputation > half lower limb |
| 1 | Gangrenous tissue > half foot |
| 2 | Gangrenous tissue > half foot, with lower limb muscle necrosis |
| 3 | Gangrenous tissue > half foot, without lower limb muscle necrosis |
| 4 | Pale foot or gait abnormalities |
| 5 | Normal |

**C)**

| **Modified ischemia score** |  |
| --- | --- |
| 0 | Auto-amputation of leg |
| 1 | Leg necrosis |
| 2 | Foot necrosis |
| 3 | Discoloration of > two toes |
| 4 | Discoloration of one toe |
| 5 | Discoloration of > two nails |
| 6 | Discoloration of one nail |
| 7 | No Necrosis |

**Bibliography**

1. Beltran-Camacho, L., et al., *Identification of the initial molecular changes in response to circulating angiogenic cells-mediated therapy in critical limb ischemia.* Stem Cell Res Ther, 2020. **11**(1): p. 106.

2. Tarlov, I.M., *Spinal cord compression studies. III. Time limits for recovery after gradual compression in dogs.* AMA Arch Neurol Psychiatry, 1954. **71**(5): p. 588-97.

3. Garcia, S., et al., *Prognostic value of 12-lead electrocardiogram and peak troponin I level after vascular surgery.* J Vasc Surg, 2013. **57**(1): p. 166-72.

4. Brenes, R.A., et al., *Toward a mouse model of hind limb ischemia to test therapeutic angiogenesis.* J Vasc Surg, 2012. **56**(6): p. 1669-79; discussion 1679.
